# Are comorbidities of patients with adrenal incidentaloma tied to sex?

**DOI:** 10.3389/fendo.2024.1385808

**Published:** 2024-05-14

**Authors:** Soraya Puglisi, Anja Barač Nekić, Valentina Morelli, Ylenia Alessi, Michele Fosci, Angelo Pani, Karin Zibar Tomsic, Serena Palmieri, Francesco Ferraù, Anna Pia, Iacopo Chiodini, Darko Kastelan, Giuseppe Reimondo, Massimo Terzolo

**Affiliations:** ^1^ Department of Clinical and Biological Sciences, Internal Medicine, San Luigi Gonzaga Hospital, University of Turin, Orbassano, Italy; ^2^ Department of Endocrinology, University Hospital Zagreb, Zagreb, Croatia; ^3^ Unit for Bone Metabolism Diseases and Diabetes and Lab of Endocrine and Metabolic Research, IRCCS, Istituto Auxologico Italiano, Milan, Italy; ^4^ Department of Human Pathology G. Barresi, Endocrine Unit, University Hospital G. Martino, University of Messina, Messina, Italy; ^5^ Department of Medical Sciences and Public Health, Endocrinology and Obesity Unit, University of Cagliari, Cagliari, Italy; ^6^ Endocrinology Unit, Fondazione Istituto di Ricovero e Cura a Carattere Scientifico (IRCCS) Ca’ Granda Ospedale Maggiore Policlinico, Milan, Italy; ^7^ Department of Biotechnology and Translational Medicine, Unit of Endocrinology, Ospedale Niguarda Cà Granda, University of Milan, Milan, Italy

**Keywords:** gender, sexual dimorphism, adrenal tumors, cortisol, MACS, subclinical Cushing, cardiovascular risk, metabolic syndrome

## Abstract

**Background:**

A recent cross-sectional study showed that both comorbidities and mortality in patients with adrenal incidentaloma (AI) are tied to sex. However, few longitudinal studies evaluated the development of arterial hypertension, hyperglycemia, dyslipidemia and bone impairment in patients with AI. The aim of this study is to analyze the impact of sex in the development of these comorbidities during long-term follow-up.

**Methods:**

We retrospectively evaluated 189 patients (120 females, 69 males) with AI, from four referral centers in Italy and Croatia. Clinical characteristics, comorbidities and cortisol after 1-mg dexamethasone suppression test (1-mg DST) were assessed at baseline and at last follow-up visit (LFUV). Median follow-up was 52 (Interquartile Range 25-86) months.

**Results:**

The rates of arterial hypertension and hyperglycemia increased over time both in females (65.8% at baseline *versus* 77.8% at LFUV, *p*=0.002; 23.7% at baseline *versus* 39.6% at LFUV, *p*<0.001; respectively) and males (58.0% at baseline *versus* 69.1% at LFUV, *p*=0.035; 33.8% at baseline *versus* 54.0% at LFUV, *p*<0.001; respectively). Patients were stratified in two groups using 1.8 µg/dl as cut-off of cortisol following 1-mg DST: non-functional adrenal tumors (NFAT) and tumors with mild autonomous cortisol secretion (MACS). In the NFAT group (99 patients, females 62.6%), at baseline, we did not observe any difference in clinical characteristics and comorbidities between males and females. At LFUV, males showed a higher frequency of hyperglycemia than females (57.6% *versus* 33.9%, *p*=0.03). In the MACS group (89 patients, females 64.0%), at baseline, the prevalence of hypertension, hyperglycemia and dyslipidemia was similar between sexes, despite females were younger (60, IQR 55-69 *versus* 67.5, IQR 61-73, years; *p*=0.01). Moreover, females presented higher rates of bone impairment (89.3% *versus* 54.5%, *p*=0.02) than males. At LFUV, a similar sex-related pattern was observed.

**Conclusion:**

Patients with AI frequently develop arterial hypertension and hyperglycemia and should be periodically checked for these comorbidities, regardless of sex. In patients with MACS, the lack of difference between sexes in the frequency of cardiometabolic comorbidities despite that females are younger, and the higher frequency of bone impairment in females, suggest a sex-specific effect of cortisol.

## 1 Introduction

It is increasingly common to deal with a patient bearing an adrenal mass in many clinical settings, from the general practitioner to various medical and surgical specialties. Due to the widespread use of cross-sectional imaging, the number of diagnoses of adrenal tumors increased by 10 times in the last decades (from 4.4 per 100.000 person-years in 1995 to 47.8 per 100.000 person-years in 2017) (1). Incidentally discovered masses (also called adrenal incidentalomas, AI), which in most of cases are benign adenomas without clinically overt hormone excess, account for most of these diagnoses.

Despite the absence of classic signs and symptoms of cortisol excess, up to 50% of patients with AI present with a mild autonomous cortisol secretion (MACS) (2, 3). This condition, previously called subclinical Cushing syndrome, is currently defined by the failure to adequately suppress cortisol after an overnight dexamethasone administration (1-mg dexamethasone suppression test, 1-mg DST) (4–6).

Patients with AI and MACS are receiving increasing attention, because of abundant evidence of higher risk of cardiometabolic morbidity and mortality (2, 3, 7–9). Surprisingly, an increased cardiometabolic risk has been reported also in patients with non-functioning adrenal tumors (NFAT) (10, 11). Although different hypotheses have been formulated to explain this phenomenon, it seems reasonable to consider cortisol secretion as a continuum instead of a dichotomic condition (12), implying that even tumors defined as nonfunctioning could still secrete cortisol in slight excess and this may be associated with type 2 diabetes mellitus (T2DM) and hypertension (13, 14).

It is also known that cortisol excess exerts detrimental effects on bone mineral density (BMD) and its microarchitecture, although this topic is still under investigation (6). Several studies have shown greater deterioration of bone quality and higher risk of vertebral fracture in patients with MACS compared to those with NFAT (15–18).

Recently, a systematic review assessed the association of MACS with increased risk for cardiometabolic comorbidities and mortality (19), and these data were used to draft the European Society of Endocrinology - European Network for the Study of Adrenal Tumors (ESE-ENSAT) clinical practice guidelines on adrenal incidentalomas (6). Interestingly, most of the 46 analyzed studies were cross-sectional, whereas only a few were longitudinal. Focusing on the development of comorbidities during a follow-up period ranging from 2 to 6.9 years, the analysis revealed that the risk of developing hypertension, T2DM and dyslipidemia was similar between MACS and NFAT (20–23). Conversely, the incidence of new vertebral fractures was higher in patients with MACS, although the database is limited to one longitudinal study (15). Another longitudinal study reported that patients with AI had a 27% higher risk of new fractures when compared to general population, but in this study the degree of cortisol secretion was unknown in 78% of patients, hampering a reliable comparison between MACS and NFAT (24).

A critical point is that only few data on sex differences are available in patients with AI (9, 25), despite the growing interest in this topic (26, 27). However, addressing sex differences in the development of comorbidities associated with AI is worth doing due to the sex-related pattern of these diseases in the general population (28–30).

The aim of this study is to analyze the impact of sex in the development of comorbidities (hypertension, hyperglycemia, dyslipidemia and bone impairment) during long-term follow-up of patients with AI.

## 2 Methods

### 2.1 Patients

Data were gathered from a retrospective cohort including 411 patients with AI who were diagnosed from 2010 to 2014 at four expert centers for adrenal diseases, three in Italy (Azienda Ospedaliero Universitaria “San Luigi Gonzaga”, Orbassano; Fondazione IRCCS Ca' Granda - Ospedale Maggiore Policlinico, Milano; Azienda Ospedaliero Universitaria “Policlinico G. Martino”, Messina) and one in Croatia (University Hospital, Zagreb). Some data of this cohort have been included in a previous study that aimed to evaluate the frequency of single nucleotide polymorphisms of the glucocorticoid receptor genes in patients with AI and their relationship with cardiometabolic comorbidities (31).

The inclusion and exclusion criteria cohort, as well as the types of data collected, have been previously reported (31). Briefly, only subjects with presumed benign cortical adenoma were included, while patients with overt Cushing syndrome, pheochromocytoma, primary aldosteronism, and patients with suspicious tumors were excluded.

**Figure 1:**
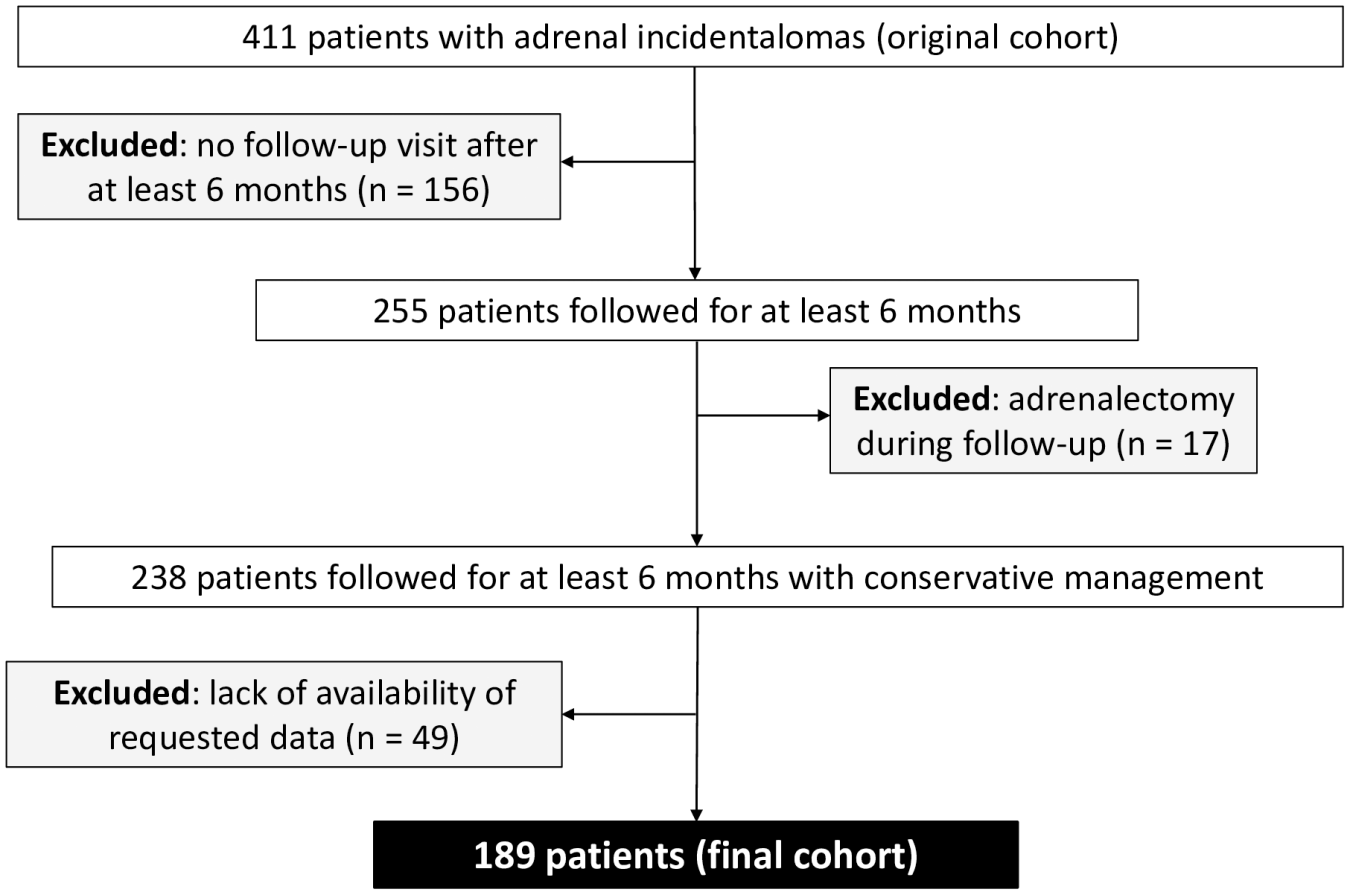
Flow diagram of study participants.

For the purpose of the present study, we included only the patients of the original cohort who have received a follow-up visit since 2014 and have a surveillance period of at least 6 months. Only patients who were managed conservatively were included, while patients who underwent adrenalectomy during follow-up were excluded (**Figure 1**).

In addition to the baseline data available from the previous study (31), we collected the following data at the last follow-up, which was extended until December 31, 2021: weight, height, body mass index (BMI), serum cortisol after overnight 1-mg DST. Moreover, we collected data both at baseline and at last follow-up about arterial hypertension, hyperglycemia, dyslipidemia and bone mineral density (BMD) reduction.

Patients with a BMI ranging from 25 to 29.9 kg/m^2^ were classified as overweight, while patients with a BMI > 30 kg/m^2^ were considered obese.

Arterial hypertension was defined by systolic blood pressure >140 mmHg and/or diastolic blood pressure >90 mmHg, or if anti-hypertensive treatment was instituted (32). Hyperglycemia included impaired fasting glucose (IFG), defined by fasting plasma glucose concentrations between 100 and 125 mg/dL (from 5.6 to 6.9 mmol/L); impaired glucose tolerance (IGT), defined by plasma glucose concentrations between 140 and 199 mg/dL (from 7.8 to 11.0 mmol/L) 2 hours after a 75-g oral load; T2DM, diagnosed when fasting blood glucose levels were 126 mg (7 mmol/L) or greater in two consecutive determinations, or at least 200 mg (11.1 mmol/L) 2 hours after an oral glucose load, or in patients treated with anti-diabetic drugs (33). Dyslipidemia included hypertriglyceridemia, diagnosed when triglyceride levels were above 1.69 mmol/L, whereas hypercholesterolemia was diagnosed when LDL cholesterol levels were above 3.36 mmol/L, or current hypolipemic medication (34). Bone impairment included osteopenia or osteoporosis, which were defined from the World Health Organization (WHO) according to BMD measurement at the spine, hip, or forearm by dual-energy x-ray absorptiomery (DXA) devices: osteopenia as a T-score between −1.0 and −2.5, osteoporosis as a T score at −2.5 or lower (35).

We established that patients should have a minimum data set at follow-up (BMI, 1-mg DST, presence of hypertension, hyperglycemia, dyslipidemia and bone impairment) to be included in the analysis.

### 2.2 Ethics

The study was performed according to the Helsinki Declaration with written informed consent was obtained from all subjects and approved by the Local Ethics Committees.

### 2.3 Statistical analysis

Continuous variables were presented using medians and interquartile ranges (IQR) and categorical variables were presented with frequencies and percentages. Differences between groups were analyzed with the Mann-Whitney test for continuous variables and the Chi-square test for categorical variables. To compare each characteristic at baseline and at last follow-up visit, we used the Wilcoxon test for continuous variables and McNemar test for categorical variables.

Given the retrospective observational nature of our study, a preliminary sample size estimation was not conducted. This approach stems from the explorative aim of analyzing the existing dataset of all available cases. This decision aimed to maximize the use of available data to enrich existing literature, focusing primarily on descriptive and exploratory objectives rather than statistical hypothesis testing, thus maintaining the study’s validity despite the absence of predetermined sample size.

All reported P values were two-sided. P-values less than 0.05 were considered as statistically significant. The statistical analyses were performed with *Jamovi - version* 2.3.18.

## 3 Results

### 3.1 Characteristics of the cohort

We included 189 patients (**Figure 1**), 120 females (F) and 69 males (M), with a median age of 61 (IQR 54-68) years at the time AI were detected. At baseline, we did not observe any significant difference in clinical characteristics and comorbidities between M and F, except for higher rates of bone impairment in F patients (76% *versus* 50%, *p* = 0.03) (**Table 1**).

**Table 1:** Sex differences in baseline characteristics.

Characteristics	Males (N= 69)	Females (N= 120)	*p value*
Age*, year	62 (56-68)	59 (53-67)	0.09
*Valid cases*	*69*	*120*	
BMI*, kg/m^2^	27.1 (25.2-30.9)	29.0 (24.6-33.3)	0.26
*Valid cases*	*64*	*109*	
**BMI category**, N (%)			0.06
Normal weight	13 (20.3%)	28 (25.7%)	
Overweight	31 (48.4%)	33 (30.3%)	
Obesity	20 (31.3%)	48 (44.0%)	
*Valid cases*	*64*	*109*	
**Hypertension**, N (%)	40 (58.0%)	79 (65.8%)	0.28
*Valid cases*	*69*	*120*	
**Hyperglycemia**, N (%)	23 (33.8%)	28 (23.7%)	0.14
*Valid cases*	*68*	*118*	
**Dyslipidemia**, N (%)	39 (56.5%)	59 (49.2%)	0.33
*Valid cases*	*69*	*120*	
**Bone impairment**, N (%)	10 (50.0%)	38 (76.0%)	**0.03**
*Valid cases*	*20*	*50*	
1-mg DST cortisol*, µg/dl	1.7 (1.2-2.6)	1.7 (1.1-2.5)	0.97
*Valid cases*	*69*	*119*

*Data are expressed as median (IQR).

BMI, body mass index; 1-mg DST cortisol, cortisol after 1 mg dexamethasone suppression test. The bold values denote statistically significant p-values at 0.05 level.

Median follow-up was 52 (IQR 25-86) months, without significant difference between sexes (49 months, IQR 20-85, for M *versus* 52 months, IQR 30-86, for F, *p* = 0.2).

### 3.2 Follow-up findings in female patients with AI

Clinical characteristics and hormonal data of the 120 female patients with AI, at baseline and at the last follow-up visit, are reported in **Supplementary Table 1**. The majority of patients were in a post-menopausal state (86.1% at baseline and 90.6% at the final follow-up visit), with none undergoing hormone replacement therapy. Among the pre-menopausal patients, none utilized hormonal contraception.

**Figure 2:**
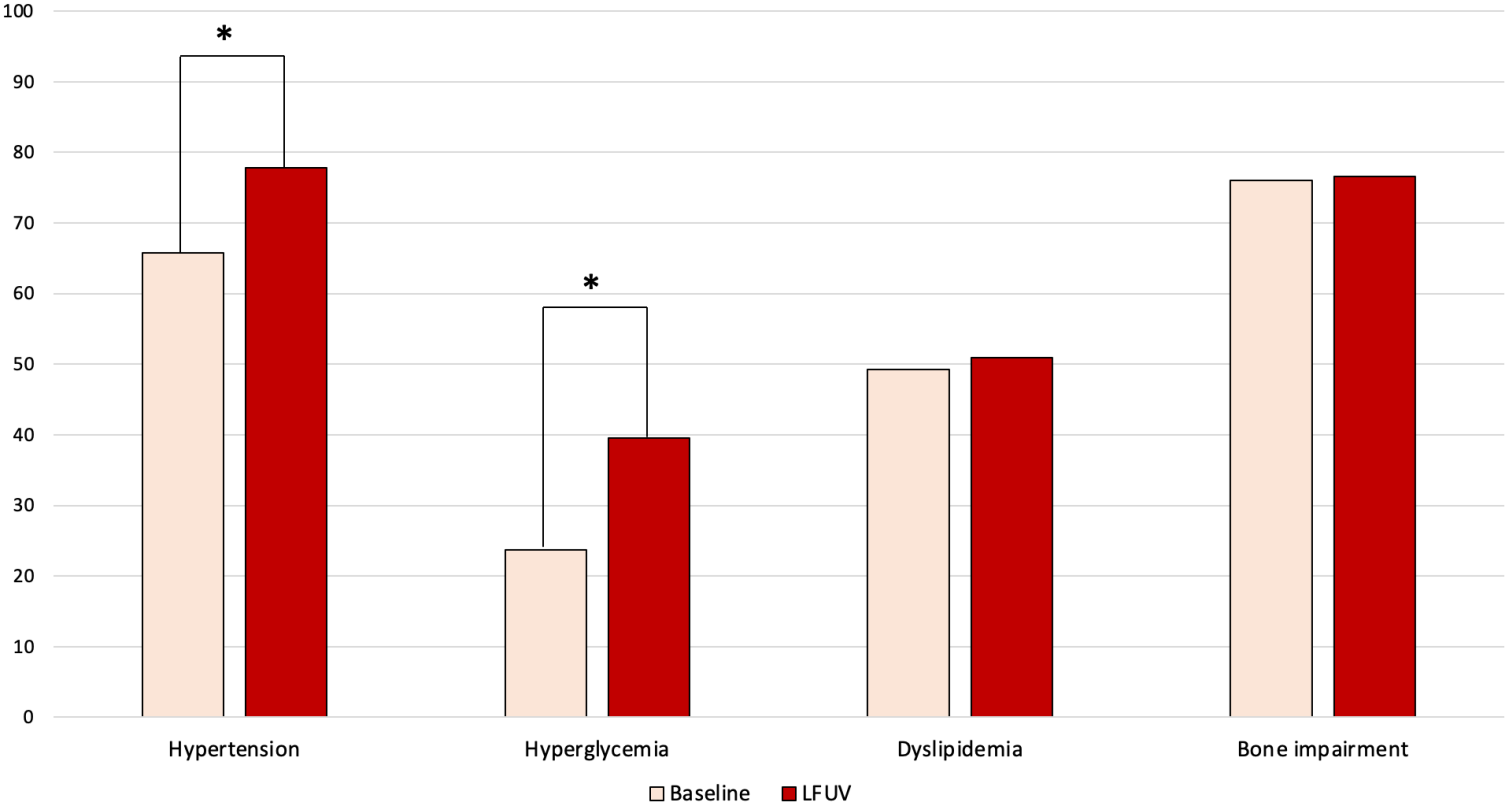
Changes over time in comorbidities of female patients with adrenal incidentaloma. LFUV, last follow-up visit. * indicates statistically significant difference (*p *< 0.05).

At last follow-up visit, arterial hypertension (77.8% *versus* 65.8%, *p* = 0.002) and hyperglycemia (39.6% *versus* 23.7%, *p* < 0.001) were more frequent than at baseline (**Figure 2**). At baseline, IGT/IFG and DM2 were present in 11 (9.3%) and in 17 (14.4%) patients, and at last follow-up visit in 22 (18.9%) and 24 (20.7%) patients, respectively (*p* < 0.001).

### 3.3 Follow-up findings in male patients with AI

**Figure 3:**
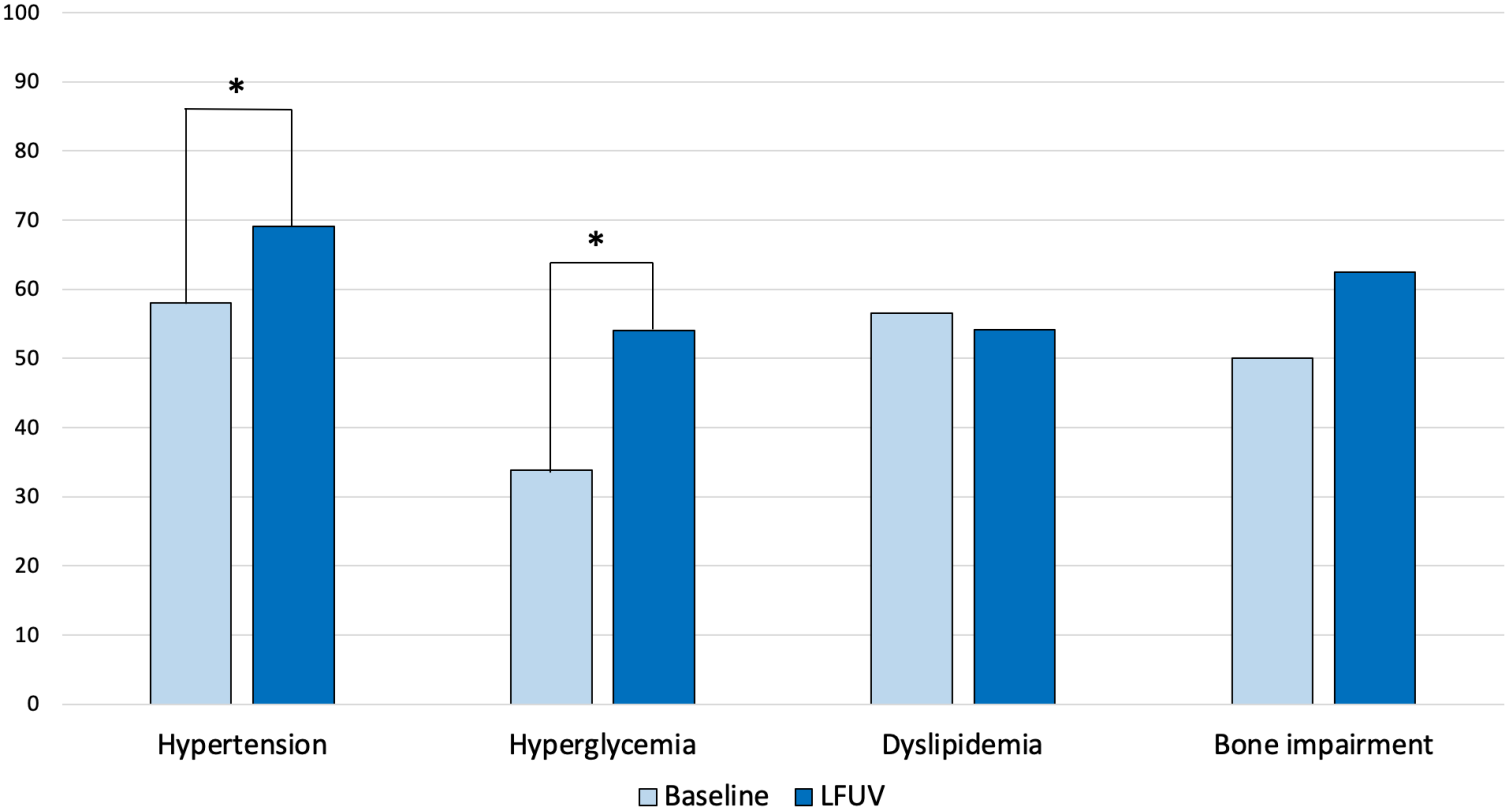
Changes over time in comorbidities of male patients with adrenal incidentaloma. LFUV, last follow-up visit. * indicates statistically significant difference (*p* < 0.05).

Clinical characteristics and hormonal data of the 69 male patients with AI, at baseline and at the last follow-up visit, are reported in **Supplementary Table 2**. At last follow-up visit, arterial hypertension (69.1% *versus* 58.0%, *p* = 0.035) and hyperglycemia (54.0% *versus* 33.8%, *p* < 0.001) were more frequent than at baseline (**Figure 3**). At baseline, IGT/IFG and DM2 were present in 14 (20.6%) and in 9 (13.2%) patients, and at last follow-up visit in 15 (23.8%) and 19 (30.2%) patients, respectively (*p* = 0.002).

### 3.4 Sex differences in groups stratified according 1-mg DST cortisol at baseline

According to the recent ESE-ENSAT guidelines (6), we used the value of cortisol following 1-mg DST >1.8 μg/dl at baseline to stratify our patients in two different groups: 99 patients with NFAT (62 F, 37 M) and 89 patients with MACS (57 F, 32 M). The 1-mg DST cortisol at baseline was unavailable only in one female patient. The distribution of sexes between the two groups was similar (62.6% F in NFAT group *versus* 64.0% in MACS groups, *p* = 0.84).

In the NFAT group, at baseline, we did not observe any significant difference in clinical characteristics and comorbidities between M and F. At last follow-up visit, a higher frequency of hyperglycemia (57.6% *versus* 33.9%, *p* = 0.03) was reported in M compared with F patients (**Table 2**).

**Table 2:** Sex-related differences among patients with non-functional adrenal tumors (NFAT) at baseline and at last follow-up visit.

NFAT	BASELINE			LAST FOLLOW-UP VISIT		
Characteristics	Women N= 62	Men N= 37	*p* *value*	Women N= 62	Men N= 37	*p* *value*
Age*, year	57 (49-65)	58 (54-64)	0.85	64 (53-71)	62 (57-69)	0.79
*Valid cases*	*62*	*37*		*62*	*37*	
BMI*, kg/m^2^	29.3 (25.0-34.3)	26.7 (25.2-30.3)	0.27	29.6 (24.7-33.5)	29.2 (25.6-32.0)	0.97
*Valid cases*	*57*	*35*		*35*	*23*	
**BMI category**, N (%)			0.07			0.27
Normal weight	14 (24.6%)	7 (20.0%)		9 (25.7%)	3 (13.0%)	
Overweight	16 (28.0%)	18 (51.4%)		10 (28.6%)	11 (47.9%)	
Obesity	27 (47.4%)	10 (28.6%)		16 (45.7%)	9 (39.1%)	
*Valid cases*	*57*	*35*		*35*	*23*	
**Hypertension**, N (%)	38 (61.3%)	19 (51.3%)	0.33	45 (75.0%)	23 (63.9%)	0.25
*Valid cases*	*62*	*37*		*60*	*36*	
**Hyperglycemia**, N (%)	14 (23.0%)	14 (38.9%)	0.09	20 (33.9%)	19 (57.6%)	**0.03**
*Valid cases*	*61*	*36*		*59*	*33*	
**Dyslipidemia**, N (%)	27 (43.5%)	23 (63.2%)	0.07	24 (42.1%)	17 (56.7%)	0.20
*Valid cases*	*62*	*37*		*57*	*30*	
**Bone impairment**, N (%)	13 (59.1%)	4 (44.4%)	0.46	27 (67.5%)	10 (66.7%)	0.95
*Valid cases*	*22*	*9*		*40*	*15*	
1-mg DST cortisol*, µg/dl	1.1 (0.9-1.4)	1.2 (0.8-1.5)	0.72	1.4 (1.0-1.9)	1.4 (1.1-1.7)	0.97
*Valid cases*	*62*	*37*		*45*	*26*	

*Data are expressed as median (IQR).

BMI, body mass index; 1-mg DST cortisol, cortisol after 1 mg dexamethasone suppression test. The bold values denote statistically significant p-values at 0.05 level.

In the MACS group, at baseline, F patients were younger (60, IQR 55-69 versus 67.5, IQR 61-73, years; *p* = 0.01) and presented higher rates of bone impairment (89.3% *versus* 54.5%, *p* = 0.02) than M patients. At last follow-up visit, the median age remained lower (66, IQR 61-73 versus 73.5, IQR 65-78, years; *p* = 0.02) and bone impairment more frequent (88.9% *versus* 58.8%, *p* = 0.01) in F patients compared with M patients (**Table 3**).

**Table 3:** Sex-related differences among patients with mild autonomous cortisol secretion (MACS) at baseline and at last follow-up visit.

MACS	BASELINE			LAST FOLLOW-UP VISIT		
Characteristics	Women N= 57	Men N= 32	*p* *value*	Women N= 57	Men N= 32	*p* *value*
Age*, year	60 (55-69)	67.5 (61-73)	**0.01**	66 (61-73)	73.5 (65-78)	**0.02**
*Valid cases*	*57*	*32*		*57*	*32*	
BMI*, kg/m^2^	28.4 (24.4-31.8)	27.3 (25.2-32.0)	0.81	27.3 (24.3-31.6)	29.1 (25.7-31.8)	0.46
*Valid cases*	*51*	*29*		*37*	*23*	
**BMI category**, N (%)			0.58			0.54
Normal weight	14 (27.5%)	6 (20.7%)		13 (35.1%)	5 (21.7%)	
Overweight	17 (33.3%)	13 (44.8%)		13 (35.1%)	10 (43.5%)	
Obesity	20 (39.2%)	10 (34.5%)		11 (29.8%)	8 (34.8%)	
*Valid cases*	*51*	*29*		*37*	*23*	
**Hypertension**, N (%)	40 (70.2%)	21 (65.6%)	0.66	45 (80.3%)	24 (75.0%)	0.56
*Valid cases*	*57*	*32*		*56*	*32*	
**Hyperglycemia**, N (%)	13 (23.2%)	9 (28.1%)	0.61	25 (44.6%)	15 (50%)	0.63
*Valid cases*	*56*	*32*		*56*	*30*	
**Dyslipidemia**, N (%)	32 (56.1%)	16 (50%)	0.58	31 (59.6%)	16 (51.6%)	0.48
*Valid cases*	*57*	*32*		*52*	*31*	
**Bone impairment**, N (%)	25 (89.3%)	6 (54.5%)	**0.02**	32 (88.9%)	10 (58.8%)	**0.01**
*Valid cases*	*28*	*11*		*36*	*17*	
1-mg DST cortisol*, µg/dl	2.5 (2.3-3.5)	2.6 (2.2-3.5)	1.00	2.7 (1.6-3.9)	2.7 (1.9-4.0)	0.80
*Valid cases*	*57*	*32*			*39*	*26*	

*Data are expressed as median (IQR).

BMI, body mass index; 1-mg DST cortisol, cortisol after 1 mg dexamethasone suppression test. The bold values denote statistically significant p-values at 0.05 level.

### 3.5 Changes over time in secretion status and their impact on the comorbidities

Among patients classified as NFAT at baseline, the 1-mg DST cortisol concentration at the last follow-up was >1.8 µg/dl in 16 patients (12 F, 4 M), whereas in 55 patients (33 F, 22 M) cortisol remained suppressed after 1-mg DST (data were available in 71 patients). We did not observe any significant difference in comorbidities at the last follow-up between these two groups, even when stratified by sex (**Supplementary Table 3A**).

Among patients classified as MACS at baseline, the 1-mg DST cortisol concentrations at the last follow-up remained >1.8 µg/dl in 47 patients (27 F, 20 M), whereas in 18 patients (12 F, 6 M) cortisol values were suppressed after 1-mg DST (data were available in 65 patients). We did not observe any significant difference in comorbidities at last the follow-up between these two groups, except for hypertension that occurred more frequently in patients with persisting lack of suppression after 1-mg DST, although no difference was present stratifying by sex (**Supplementary Table 3B**).

## 4 Discussion

At the best of our knowledge, this is the first study that did a comprehensive assessment of the sex-related patterns in the development of comorbidities (hypertension, hyperglycemia, dyslipidemia and bone impairment) during long-term follow-up of patients with AI, allowing several considerations.

First, we found that both female and male patients with AI frequently developed arterial hypertension and hyperglycemia during follow-up, despite no significant changes in the degree of cortisol autonomy secretion. In some patients, we observed a change in the secretory status from NFAT to MACS, or vice versa, but numbers were too small for a meaningful assessment. A possible explanation for these changes could be the concomitant use of drugs that influence 1-mg DST cortisol levels. As a matter of fact, although we excluded the main confounders (i.e. no patient assumed estrogens), due to the retrospective design of our study it is challenging to ascertain the effect of all possible medications that could affect the dexamethasone metabolism (36, 37).

Our findings reinforce the recommendations of the ECE-ENSAT guidelines (6) of avoiding periodic reassessment of cortisol secretion in specialized endocrine centers, suggesting instead a clinical follow-up that should be done at the level of primary health care (6). However, considering that a percentage of patients with AI may show variations in their secretory activity and imaging characteristics over time, it is crucial to pursue a tailored follow-up strategy, although the optimal frequency and duration of follow-up is still a matter of debate (38).

A second important point is that, given the age-related pattern of hypertension and hyperglycemia (39, 40), in the MACS group there was no significant difference between sexes in the rates of these comorbidities, despite females were younger than males both at baseline and at last follow-up. Therefore, it could be assumed that women may be more prone to develop comorbidities potentially linked to cortisol excess in younger age than men. This finding is in line with the results of a recent cross-sectional large multicenter study including 4374 patients with AI, which showed a significant increase of all-cause mortality and major adverse cardiovascular event (MACE) in female patients younger than 65 years with autonomous cortisol secretion (9). Moreover, a monocentric study including 98 patients with MACS reported only in premenopausal women a direct correlation between autonomous cortisol secretion levels and levels of fasting C-peptide or fasting C-peptide-to-glucose ratio, which are considered indexes of insulin resistance (41).

In the last decades, the differences between sexes in the impact of cortisol excess on comorbidities has been explored in patients with endogenous hypercortisolism of pituitary origin (Cushing disease, CD), males presented at diagnosis with more frequent or more severe comorbidities (42, 43). Pecori Giraldi et al. reported higher prevalence of osteoporosis in men, without difference in the occurrence of glucose intolerance or T2DM, hypertension, and obesity (42). Zilio et al. reported higher rates of clinically evident fractures and lumbar osteoporosis in males than in females at diagnosis (43). Moreover, using a logistic regression model including age, sex, BMI, hypogonadism and values of urinary free cortisol, male gender was found to be associated with hypertriglyceridemia, low levels of HDL, number of antihypertensive drugs, lumbar osteoporosis and fractures (43). In a systematic review, male sex has been found to be a predictive factor of cardiovascular events and mortality in CD (44). Conversely, in the setting of exogenous hypercortisolism, women have been found to be more prone to develop metabolic and bone impairment as a result of chronic glucocorticoid treatment (45–48). Moreover, oral corticosteroid treatment in patients with chronic obstructive pulmonary disease during long-term oxygen therapy has been associated with increased mortality in women but not in men (49, 50).

A recent review on the sexual dimorphism of glucocorticoid signaling suggests that this discrepancy in outcomes can be due to differences in androgen levels between sexes in the different settings mentioned above. Since glucocorticoid treatment suppresses the secretion of adrenocorticotropin hormone (ACTH) via negative feedback, adrenal ACTH-dependent androgens are lower in these patients. Conversely, in patients with ACTH-dependent Cushing syndrome, adrenal androgen secretion is stimulated. The authors suggest that androgens interact with cortisol to foster development of comorbidities in male patients with CD, whereas in patients treated with exogenous glucocorticoids other mechanisms may be operative (51).

Unfortunately, the complex interplay between sex hormones and the hypothalamic-pituitary-adrenal (HPA) axis has been only minimally understood. It is recognized that from early life to adulthood, androgens and estrogens differentially affect the HPA axis, leading to sex differences into activation and regulation and partially explaining the different responsivity to stress condition. However, the results of the studies in this field, which in most cases have been carried out in mice and not in humans, resulted controversial and difficult to interpret in a clinically meaningful way (52).

The cross-talk between HPA axis and gonadal hormones in stress responsivity could be particularly relevant in consideration of the key role of stress in the pathogenesis of cardiovascular diseases. In the general population, a strong association between mental stress and cardiovascular disease has been recognized (53). Interestingly, young women seem to have a higher risk of developing cardiovascular events in response to stress compared to age-matched men (54). Given the role of the HPA axis in stress conditions, a possible explanation for this sexual dimorphism is an increased activation of the signaling of the glucocorticoid receptors in women. It should be also considered the sexual dysmorphism in the anti-inflammatory effects of glucocorticoids, given the different response to inflammation in females and males demonstrated in several autoimmune diseases (55). Similar mechanisms could explain the higher susceptibility in women with AI to early develop cardiometabolic comorbidities when exposed to mild but prolonged excess of cortisol. It could be assumed that the discrepancy in the sex dysmorphism of cardiometabolic and bone alterations between patients with ACTH-dependent Cushing syndrome and patients with MACS could be due to the different lengths of exposure to cortisol excess, and also to the different interactions of the HPA axis with the gonadal hormones, in these different settings.

The interplay between HPA axis and sex hormones could also explains the higher occurrence of bone impairment that we found in women with MACS. This finding aligns with broader epidemiological data indicating that women are generally at a higher risk for osteoporosis and related bone health issues compared to men (56, 57). Several factors contribute to this disparity, both in the general population and potentially within our cohort. Particularly, the hormonal changes that accompany menopause can significantly impact bone density. Given the age distribution and the high percentage of women in post-menopausal status in our cohort, it is plausible that the observed sex differences in bone health may be partly attributable to the decline in estrogens levels which play a pivotal role in the acceleration of bone loss in women. However, evidence from literature seem suggest that also the presence of AI may exert differential effects on bone metabolism between sexes. A higher prevalence of fractures has been reported by Zavatta et al. in postmenopausal women with MACS compared with postmenopausal women with NFAT, whereas no difference was found in men with AI stratified according to autonomous cortisol secretion (25). Similarly, a previous study including 70 female patients with AI reported higher prevalence of vertebral fractures in postmenopausal women with MACS than with NFAT (58). In both studies, a regression analysis showed that MACS predicted fractures only in postmenopausal women (25, 58). These results are in line with ours, corroborating the hypothesis of a cross-talk between HPA axis and gonadal hormones resulting in the amplification of the sex-related bone impairment in patients with AI subject to chronic exposure to mild excess of cortisol. Interestingly, a recent study demonstrated a higher prevalence of vertebral fractures also in male patients with MACS than with NFAT, although the authors partially justified these conflicting results with a possible higher disease activity in their cohort (18). It is important to consider, however, that in the condition of glucocorticoid excess the BMD determination is not enough sensitive to identify patients with bone impairment. Indeed, these patients experience fractures often in the presence of a normal or only slightly reduced BMD, since the bone quality reduction rather than the bone density decrease is the main determinant of bone fragility in the condition of hypercortisolism (59). Therefore, considering the lack of data on fracture risk in our cohort, the present results on the gender differences in bone impairment in AI patients have to be considered with caution.

The main limitations of our study are the retrospective nature and the limited sample size. Given the retrospective nature of the study, detailed data on sex hormones levels, cardiovascular events, and the treatment of comorbidities were not adequately available for inclusion in the analysis. Moreover, the need to have the availability of a DXA analysis to evaluate the bone impairment, which was performed selectively and not across the entire cohort, introduced a potential bias of overdiagnosis, especially in female patients. The decision to perform DXA scans selectively was influenced by clinical guidelines and the feasibility within the context of our study’s design. However, we acknowledge that this approach may not fully capture the prevalence and severity of bone health issues across all individuals with AI. Specifically, the absence of universal DXA screening may overlook milder forms of bone impairment in patients not subjected to this analysis, thereby inflating the perceived incidence of osteoporosis among those tested, particularly among women known to be at higher risk for such conditions. This limitation underscores the necessity for cautious interpretation of our findings regarding bone health disparities by gender among AI patients.

An additional important limitation is the absence of a comparison group from the general population, which indeed makes it challenging to conclusively assert that the worsening of arterial hypertension and hyperglycemia was specifically due to the AI presence instead of being merely a reflection of the natural aging process. However, studies have shown that patients with AI exhibited a higher prevalence of these comorbidities compared to age-matched controls without AI (60–63), supporting the notion that AI may contribute to these outcomes more than age alone. Future research should aim to incorporate a comparison to more definitively ascertain the specific impact of AI on the development of cardiometabolic complications. Until then, our findings add to a growing body of evidence suggesting a link between AI and these comorbidities, warranting further investigation and consideration in clinical practice.

Strengths of our study include data collection in different referral center for adrenal diseases and the length of follow-up.

In conclusion, our findings support the hypothesis of a sex-specific effect of cortisol in the development of comorbidities resulting in higher risk of bone impairment and occurrence of cardiometabolic complications at younger age for women with MACS.

## Data availability statement

The original contributions presented in the study are included in the article/**Supplementary Material**. Further inquiries can be directed to the corresponding author.

## Ethics statement

The studies involving humans were approved by San Luigi Hospital, Orbassano (Turin). The studies were conducted in accordance with the local legislation and institutional requirements. The participants provided their written informed consent to participate in this study.

## Author contributions

SPu: Conceptualization, Data curation, Formal analysis, Investigation, Methodology, Validation, Visualization, Writing – original draft, Writing – review & editing. AN: Data curation, Investigation, Writing – original draft, Writing – review & editing. VM: Data curation, Investigation, Writing – original draft, Writing – review & editing. YA: Data curation, Investigation, Writing – original draft, Writing – review & editing. MF: Data curation, Investigation, Writing – original draft, Writing – review & editing. APa: Data curation, Investigation, Writing – original draft, Writing – review & editing. KT: Data curation, Investigation, Writing – original draft, Writing – review & editing. SPa: Data curation, Investigation, Writing – original draft, Writing – review & editing. FF: Data curation, Investigation, Writing – original draft, Writing – review & editing. APi: Data curation, Investigation, Writing – original draft, Writing – review & editing. IC: Data curation, Investigation, Writing – original draft, Writing – review & editing. DK: Data curation, Investigation, Writing – original draft, Writing – review & editing. GR: Funding acquisition, Resources, Supervision, Writing – original draft, Writing – review & editing. MT: Funding acquisition, Resources, Supervision, Writing – original draft, Writing – review & editing.

## References

[1] Ebbehoj A Li D Kaur RJ Zhang C Singh S Li T. Epidemiology of adrenal tumours in Olmsted County, Minnesota, USA: a population-based cohort study. Lancet Diabetes Endocrinol. (2020) 8:894–902. doi: 10.1016/S2213-8587(20)30314-4 33065059 PMC7601441

[2] Reimondo G Castellano E Grosso M Priotto R Puglisi S Pia A. Adrenal incidentalomas are tied to increased risk of diabetes: findings from a prospective study. J Clin Endocrinol Metab. (2020) 105(4):e973–81. doi: 10.1210/clinem/dgz284 31900474

[3] Prete A Subramanian A Bancos I Chortis V Tsagarakis S Lang K. Cardiometabolic disease burden and steroid excretion in benign adrenal tumors : A cross-sectional multicenter study. Ann Intern Med. (2022) 175:325–34. doi: 10.7326/M21-1737 34978855

[4] Reimondo G Puglisi S Pia A Terzolo M. Autonomous hypercortisolism: definition and clinical implications. Minerva Endocrinol. (2019) 44:33–42. doi: 10.23736/S0391-1977.18.02884-5 29963828

[5] Bancos I Prete A. Approach to the patient with adrenal incidentaloma. J Clin Endocrinol Metab. (2021) 106:3331–53. doi: 10.1210/clinem/dgab512 PMC8530736 34260734

[6] Fassnacht M Tsagarakis S Terzolo M Tabarin A Sahdev A Newell-Price J. European Society of Endocrinology clinical practice guidelines on the management of adrenal incidentalomas, in collaboration with the European Network for the Study of Adrenal Tumors. Eur J Endocrinol. (2023) 189:G1–G42. doi: 10.1093/ejendo/lvad066 37318239

[7] Di Dalmazi G Vicennati V Garelli S Casadio E Rinaldi E Giampalma E. Cardiovascular events and mortality in patients with adrenal incidentalomas that are either non-secreting or associated with intermediate phenotype or subclinical Cushing's syndrome: a 15-year retrospective study. Lancet Diabetes Endocrinol. (2014) 2:396–405. doi: 10.1016/S2213-8587(13)70211-0 24795253

[8] Debono M Bradburn M Bull M Harrison B Ross RJ Newell-Price J. Cortisol as a marker for increased mortality in patients with incidental adrenocortical adenomas. J Clin Endocrinol Metab. (2014) 99:4462–70. doi: 10.1210/jc.2014-3007 PMC4255126 25238207

[9] Deutschbein T Reimondo G Di Dalmazi G Bancos I Patrova J Vassiliadi DA. Age-dependent and sex-dependent disparity in mortality in patients with adrenal incidentalomas and autonomous cortisol secretion: an international, retrospective, cohort study. Lancet Diabetes Endocrinol. (2022) 10:499–508. doi: 10.1016/S2213-8587(22)00100- 35533704 PMC9679334

[10] Lopez D Luque-Fernandez MA Steele A Adler GK Turchin A Vaidya A. "Nonfunctional" Adrenal tumors and the risk for incident diabetes and cardiovascular outcomes: A cohort study. Ann Intern Med. (2016) 165:533–42. doi: 10.7326/M16-0547 PMC5453639 27479926

[11] Elhassan YS Alahdab F Prete A Delivanis DA Khanna A Prokop L. Natural history of adrenal incidentalomas with and without mild autonomous cortisol excess: A systematic review and meta-analysis. Ann Intern Med 171(2):107–16. (2019). doi: 10.7326/M18-3630 31234202

[12] Terzolo M Reimondo G. Insights on the natural history of adrenal incidentalomas. Ann Intern Med. (2019) 171(2):135–6. doi: 10.7326/M19-1482 31234201

[13] Arruda M Mello Ribeiro Cavalari E Pessoa de Paula M Fernandes Cordeiro de Morais F Furtado Bilro G Alves Coelho MC. The presence of nonfunctioning adrenal incidentalomas increases arterial hypertension frequency and severity, and is associated with cortisol levels after dexamethasone suppression test. J Hum Hypertens. (2017) 32:3–11. doi: 10.1038/s41371-017-0011-4 29176595

[14] Favero V Aresta C Parazzoli C Cairoli E Eller-Vainicher C Palmieri S. The degree of cortisol secretion is associated with diabetes mellitus and hypertension in patients with nonfunctioning adrenal tumors. Cardiovasc Diabetol. (2023) 22:102. doi: 10.1186/s12933-023-01836-1 37131218 PMC10155432

[15] Morelli V Eller-Vainicher C Salcuni AS Coletti F Iorio L Muscogiuri G. Risk of new vertebral fractures in patients with adrenal incidentaloma with and without subclinical hypercortisolism: a multicenter longitudinal study. J Bone Miner Res. (2011) 26:1816–21. doi: 10.1002/jbmr.398 21472775

[16] Vinolas H Grouthier V Mehsen-Cetre N Boisson A Winzenrieth R Schaeverbeke T. Assessment of vertebral microarchitecture in overt and mild Cushing's syndrome using trabecular bone score. Clin Endocrinol (Oxf). (2018) 89:148–54. doi: 10.1111/cen.13743 29781519

[17] Moraes AB de Paula MP de Paula Paranhos-Neto F Cavalari EMR de Morais FFC Curi DSC. Bone evaluation by high-resolution peripheral quantitative computed tomography in patients with adrenal incidentaloma. J Clin Endocrinol Metab. (2020) 105:e2726–37. doi: 10.1210/clinem/dgaa263 32413110

[18] Favero V Eller-Vainicher C Morelli V Cairoli E Salcuni AS Scillitani A. Increased risk of vertebral fractures in patients with mild autonomous cortisol secretion. J Clin Endocrinol Metab. (2024) 109:e623–e32. doi: 10.1210/clinem/dgad560 PMC10795935 37738555

[19] Pelsma ICM Fassnacht M Tsagarakis S Terzolo M Tabarin A Sahdev A. Comorbidities in mild autonomous cortisol secretion and the effect of treatment: systematic review and meta-analysis. Eur J Endocrinol. (2023) 189:S88–S101. doi: 10.1093/ejendo/lvad134 37801655

[20] Giordano R Marinazzo E Berardelli R Picu A Maccario M Ghigo E. Long-term morphological, hormonal, and clinical follow-up in a single unit on 118 patients with adrenal incidentalomas. Eur J Endocrinol. (2010) 162:779–85. doi: 10.1530/EJE-09-0957 20103607

[21] Morelli V Reimondo G Giordano R Della Casa S Policola C Palmieri S. Long-term follow-up in adrenal incidentalomas: an Italian multicenter study. J Clin Endocrinol Metab. (2014) 99:827–34. doi: 10.1210/jc.2013-3527 24423350

[22] Papanastasiou L Alexandraki KI Androulakis II Fountoulakis S Kounadi T Markou A. Concomitant alterations of metabolic parameters, cardiovascular risk factors and altered cortisol secretion in patients with adrenal incidentalomas during prolonged follow-up. Clin Endocrinol (Oxf). (2017) 86:488–98. doi: 10.1111/cen.13294 27992961

[23] Araujo-Castro M Robles Lazaro C Parra Ramirez P Cuesta Hernandez M Sampedro Nunez MA Marazuela M. Cardiometabolic profile of non-functioning and autonomous cortisol-secreting adrenal incidentalomas. Is the cardiometabolic risk similar or are there differences? Endocrine. (2019) 66:650–9. doi: 10.1007/s12020-019-02066-w 31473918

[24] Li D Kaur RJ Zhang CD Ebbehoj A Singh S Atkinson EJ. Risk of bone fractures after the diagnosis of adrenal adenomas: a population-based cohort study. Eur J Endocrinol. (2021) 184:597–606. doi: 10.1530/EJE-20-1396 33606665 PMC7974392

[25] Zavatta G Vicennati V Altieri P Tucci L Colombin G Coscia K. Mild autonomous cortisol secretion in adrenal incidentalomas and risk of fragility fractures: a large cross-sectional study. Eur J Endocrinol. (2023) 188:343–52. doi: 10.1093/ejendo/lvad038 36952249

[26] Baggio G Corsini A Floreani A Giannini S Zagonel V. Gender medicine: a task for the third millennium. Clin Chem Lab Med. (2013) 51:713–27. doi: 10.1515/cclm-2012-0849 23515103

[27] Mauvais-Jarvis F Bairey Merz N Barnes PJ Brinton RD Carrero JJ DeMeo DL. Sex and gender: modifiers of health, disease, and medicine. Lancet. (2020) 396:565–82. doi: 10.1016/S0140-6736(20)31561-0 PMC7440877 32828189

[28] Kautzky-Willer A Harreiter J Pacini G. Sex and gender differences in risk, pathophysiology and complications of type 2 diabetes mellitus. Endocr Rev. (2016) 37:278–316. doi: 10.1210/er.2015-1137 27159875 PMC4890267

[29] Alswat KA. Gender disparities in osteoporosis. J Clin Med Res. (2017) 9:382–7. doi: 10.14740/jocmr2970w PMC5380170 28392857

[30] Clayton JA Gaugh MD. Sex as a biological variable in cardiovascular diseases: JACC focus seminar 1/7. J Am Coll Cardiol. (2022) 79:1388–97. doi: 10.1016/j.jacc.2021.10.050 35393021

[31] Reimondo G Chiodini I Puglisi S Pia A Morelli V Kastelan D. Analysis of BCLI, N363S and ER22/23EK polymorphisms of the glucocorticoid receptor gene in adrenal incidentalomas. PLoS One. (2016) 11:e0162437. doi: 10.1371/journal.pone.0162437 27649075 PMC5029814

[32] Williams B Mancia G Spiering W Agabiti Rosei E Azizi M Burnier M. 2018 Practice Guidelines for the management of arterial hypertension of the European Society of Hypertension and the European Society of Cardiology: ESH/ESC Task Force for the Management of Arterial Hypertension. J Hypertens. (2018) 36:2284–309. doi: 10.1097/HJH.0000000000001961 30379783

[33] American diabetes A. 2. Classification and diagnosis of diabetes: Standards of medical care in diabetes-2021. Diabetes Care. (2021) 44:S15–33. doi: 10.2337/dc21-S002 33298413

[34] National Cholesterol Education Program Expert Panel on Detection E Treatment of High Blood Cholesterol in A. Third Report of the National Cholesterol Education Program (NCEP) Expert Panel on Detection, Evaluation, and Treatment of High Blood Cholesterol in Adults (Adult Treatment Panel III) final report. Circulation. (2002) 106:3143–421. doi: 10.1161/circ.106.25.3143 12485966

[35] Kanis JA Melton LJ 3rd Christiansen C Johnston CC Khaltaev N. The diagnosis of osteoporosis. J Bone Miner Res. (1994) 9:1137–41. doi: 10.1002/jbmr.5650090802 7976495

[36] Ceccato F Barbot M Scaroni C Boscaro M. Frequently asked questions and answers (if any) in patients with adrenal incidentaloma. J Endocrinol Invest. (2021) 44:2749–63. doi: 10.1007/s40618-021-01615-3 PMC8572215 34160793

[37] Valassi E Swearingen B Lee H Nachtigall LB Donoho DA Klibanski A. Concomitant medication use can confound interpretation of the combined dexamethasone-corticotropin releasing hormone test in Cushing's syndrome. J Clin Endocrinol Metab. (2009) 94:4851–9. doi: 10.1210/jc.2009-1500 PMC2795659 19850679

[38] Ceccato F Tizianel I Voltan G Maggetto G Merante Boschin I Quaia E. Attenuation value in adrenal incidentalomas: A longitudinal study. Front Endocrinol (Lausanne). (2021) 12:794197. doi: 10.3389/fendo.2021.794197 34925247 PMC8678594

[39] Collaboration NCDRF. Long-term and recent trends in hypertension awareness, treatment, and control in 12 high-income countries: an analysis of 123 nationally representative surveys. Lancet. (2019) 394:639–51. doi: 10.1016/S0140-6736(19)31145-6 PMC6717084 31327564

[40] Khan MAB Hashim MJ King JK Govender RD Mustafa H Al Kaabi J. Epidemiology of type 2 diabetes - global burden of disease and forecasted trends. J Epidemiol Glob Health. (2020) 10:107–11. doi: 10.2991/jegh.k.191028.001 PMC7310804 32175717

[41] Ouyang R Yin Y Wang J Su W Zang L Chen K. Sex differences in hypercortisolism and glucose-metabolism disturbances in patients with mild autonomous cortisol secretion: Findings From a Single center in China. Front Endocrinol (Lausanne). (2022) 13:857947. doi: 10.3389/fendo.2022.857947 35757395 PMC9218075

[42] Pecori Giraldi F Moro M Cavagnini F Study Group on the Hypothalamo-Pituitary-Adrenal Axis of the Italian Society of E. Gender-related differences in the presentation and course of Cushing's disease. J Clin Endocrinol Metab. (2003) 88:1554–8. doi: 10.1210/jc.2002-021518 12679438

[43] Zilio M Barbot M Ceccato F Camozzi V Bilora F Casonato A. Diagnosis and complications of Cushing's disease: gender-related differences. Clin Endocrinol (Oxf). (2014) 80:403–10. doi: 10.1111/cen.12299 23889360

[44] Puglisi S Perini AME Botto C Oliva F Terzolo M. Long-term consequences of cushing's syndrome: A systematic literature review. J Clin Endocrinol Metab. (2023) 109(3):e901–19. doi: 10.1210/clinem/dgad453 37536275

[45] Savas M Muka T Wester VL van den Akker ELT Visser JA Braunstahl GJ. Associations between systemic and local corticosteroid use with metabolic syndrome and body mass index. J Clin Endocrinol Metab. (2017) 102:3765–74. doi: 10.1210/jc.2017-01133 28973553

[46] Marystone JF Barrett-Connor EL Morton DJ. Inhaled and oral corticosteroids: their effects on bone mineral density in older adults. Am J Public Health. (1995) 85:1693–5. doi: 10.2105/AJPH.85.12.1693 PMC1615730 7503347

[47] Shah SH Johnston TD Jeon H Ranjan D. Effect of chronic glucocorticoid therapy and the gender difference on bone mineral density in liver transplant patients. J Surg Res. (2006) 135:238–41. doi: 10.1016/j.jss.2006.04.032 16872635

[48] Fu J Cuppen BV Welsing PM van Wietmarschen H Harms AC Berger R. Differences between serum polar lipid profiles of male and female rheumatoid arthritis patients in response to glucocorticoid treatment. Inflammopharmacology. (2016) 24:397–402. doi: 10.1007/s10787-016-0284-1 27682325 PMC5119840

[49] Strom K. Survival of patients with chronic obstructive pulmonary disease receiving long-term domiciliary oxygen therapy. Am Rev Respir Dis. (1993) 147:585–91. doi: 10.1164/ajrccm/147.3.585 8442591

[50] Strom K. Oral corticosteroid treatment during long-term oxygen therapy in chronic obstructive pulmonary disease: a risk factor for hospitalization and mortality in women. Respir Med. (1998) 92:50–6. doi: 10.1016/S0954-6111(98)90032-4 9519225

[51] Kroon J Pereira AM Meijer OC. Glucocorticoid sexual dimorphism in metabolism: Dissecting the Role of Sex Hormones. Trends Endocrinol Metab. (2020) 31:357–67. doi: 10.1016/j.tem.2020.01.010 32037025

[52] Dearing C Handa RJ Myers B. Sex differences in autonomic responses to stress: implications for cardiometabolic physiology. Am J Physiol Endocrinol Metab. (2022) 323:E281–E9. doi: 10.1152/ajpendo.00058.2022 PMC9448273 35793480

[53] Kivimaki M Steptoe A. Effects of stress on the development and progression of cardiovascular disease. Nat Rev Cardiol. (2018) 15:215–29. doi: 10.1038/nrcardio.2017.189 29213140

[54] Sullivan S Hammadah M Wilmot K Ramadan R Pearce BD Shah A. Young Women With Coronary Artery Disease Exhibit Higher Concentrations of interleukin-6 at baseline and in response to mental stress. J Am Heart Assoc. (2018) 7:e010329. doi: 10.1161/JAHA.118.010329 30571600 PMC6405549

[55] Quinn M Ramamoorthy S Cidlowski JA. Sexually dimorphic actions of glucocorticoids: beyond chromosomes and sex hormones. Ann N Y Acad Sci. (2014) 1317:1–6. doi: 10.1111/nyas.12425 24739020 PMC12455521

[56] Cavalli L Guazzini A Cianferotti L Parri S Cavalli T Metozzi A. Prevalence of osteoporosis in the Italian population and main risk factors: results of BoneTour Campaign. BMC Musculoskelet Disord. (2016) 17:396. doi: 10.1186/s12891-016-1248-8 27639376 PMC5027125

[57] Cummings SR Melton LJ. Epidemiology and outcomes of osteoporotic fractures. Lancet. (2002) 359:1761–7. doi: 10.1016/S0140-6736(02)08657-9 12049882

[58] Chiodini I Guglielmi G Battista C Carnevale V Torlontano M Cammisa M. Spinal volumetric bone mineral density and vertebral fractures in female patients with adrenal incidentalomas: the effects of subclinical hypercortisolism and gonadal status. J Clin Endocrinol Metab. (2004) 89:2237–41. doi: 10.1210/jc.2003-031413 15126547

[59] Canalis E Mazziotti G Giustina A Bilezikian JP. Glucocorticoid-induced osteoporosis: pathophysiology and therapy. Osteoporos Int. (2007) 18:1319–28. doi: 10.1007/s00198-007-0394-0 17566815

[60] Garrapa GG Pantanetti P Arnaldi G Mantero F Faloia E. Body composition and metabolic features in women with adrenal incidentaloma or Cushing's syndrome. J Clin Endocrinol Metab. (2001) 86:5301–6. doi: 10.1210/jcem.86.11.8059 11701696

[61] Androulakis II Kaltsas GA Kollias GE Markou AC Gouli AK Thomas DA. Patients with apparently nonfunctioning adrenal incidentalomas may be at increased cardiovascular risk due to excessive cortisol secretion. J Clin Endocrinol Metab. (2014) 99:2754–62. doi: 10.1210/jc.2013-4064 24712565

[62] Akkan T Altay M Unsal Y Dagdeviren M Beyan E. Nonfunctioning adrenal incidentaloma affecting central blood pressure and arterial stiffness parameters. Endocrine. (2017) 58:513–20. doi: 10.1007/s12020-017-1439-6 29043559

[63] Rebelo JFD Costa JM Junqueira FD Fonseca AO de Almeida A Moraes AB. Adrenal incidentaloma: Do patients with apparently nonfunctioning mass or autonomous cortisol secretion have similar or different clinical and metabolic features? Clin Endocrinol (Oxf). (2023) 98:662–9. doi: 10.1111/cen.14861 36514987

