# Supplementary material for: Are comorbidities of patients with adrenal incidentaloma tied to sex?
Source: Front Endocrinol (Lausanne). 2024 May 14;15:1385808. doi: 10.3389/fendo.2024.1385808 (PMC11130385; doi:10.3389/fendo.2024.1385808)
Supplement: Supplementary file 1 [file Table_1.docx]

Supplementary Material

**Supplemental Table 1.** Clinical characteristics and hormonal data of 120 female patients with adrenal incidentaloma, at baseline and at last follow-up visit.

| **Characteristics** | **Baseline** | **Last follow-up visit** | ***p value*** |
| --- | --- | --- | --- |
| **Age***, year | 59 (53-67) | 65 (59-71) | **< 0.001** |
| *Valid cases* | *120* | *120* |  |
| **Post-menopausal status,** N (%) | 99 (86.1%) | 106 (90.6%) | **0.025** |
| *Valid cases* | *115* | *117* |  |
| **BMI***, kg/m^2^ | 29.0 (24.6-33.2) | 29.0 (24.0-33.0) | 0.23 |
| *Valid cases* | *109* | *73* |  |
| **BMI category,** N (%) |  |  | 0.76 |
| Normal weight | 28 (25.7%) | 22 (30.1%) |  |
| Overweight | 33 (30.3%) | 23 (31.5%) |  |
| Obesity | 48 (44.0%) | 28 (38.4%) |  |
| *Valid cases* | *109* | *73* |  |
| **Hypertension**, N (%) | 79 (65.8%) | 91 (77.8%) | **0.002** |
| *Valid cases* | *120* | *117* |  |
| **Hyperglycemia**, N (%) | 28 (23.7%) | 46 (39.6%) | **< 0.001** |
| *Valid cases* | *118* | *116* |  |
| **Dyslipidemia**, N (%) | 59 (49.2%) | 56 (50.9%) | 0.68 |
| *Valid cases* | *120* | *110* |  |
| **Bone impairment**, N (%) | 38 (76.0%) | 59 (76.6%) | 0.08 |
| *Valid cases* | *50* | *77* |  |
| **1mg DST cortisol*,** µg/dl  *Valid cases* | 1.7 (1.1-2.5)  *119* | 1.7 (1.1-2.7)  *85* | 0.20 |

**Data are expressed as median (IQR).*

*BMI = body mass index; 1mgDST cortisol = cortisol after 1 mg dexamethasone suppression test.*

**Supplemental Table 2.** Clinical characteristics and hormonal data of 69 male patients with adrenal incidentaloma, at baseline and at last follow-up visit.

| **Characteristics** | **Baseline** | **Last follow-up visit** | ***p value*** |
| --- | --- | --- | --- |
| **Age***, year | 62 (56-68) | 66 (60-75) | **< 0.001** |
| *Valid cases* | *69* | *69* |  |
| **BMI***, kg/m^2^ | 27.1 (25.2-30.9) | 29.1 (25.6-31.7) | 0.45 |
| *Valid cases* | *64* | *46* |  |
| **BMI category**, N (%) |  |  | 0.65 |
| Normal weight  Overweight | 13 (20.3%)  31 (48.4%) | 8 (17.4%)  21 (45.7%) |  |
| Obesity | 20 (31.3%) | 17 (36.9%) |  |
| *Valid cases* | *64* | *46* |  |
| **Hypertension**, N (%) | 40 (58.0%) | 47 (69.1%) | **0.035** |
| *Valid cases* | *69* | *68* |  |
| **Hyperglycemia**, N (%) | 23 (33.8%) | 34 (54.0%) | **0.005** |
| *Valid cases* | *68* | *63* |  |
| **Dyslipidemia**, N (%) | 39 (56.5%) | 33 (54.1%) | 0.13 |
| *Valid cases* | *69* | *61* |  |
| **Bone impairment**, N (%) | 10 (50%) | 20 (62.5%) | 0.32 |
| *Valid cases* | *20* | *32* |  |
| **1mg DST cortisol*,** µg/dl  *Valid cases* | 1.7 (1.2-2.6)  *69* | 1.7 (1.4-2.6)  *52* | 0.30 |

**Data are expressed as median (IQR).*

*BMI = body mass index; 1mgDST cortisol = cortisol after 1 mg dexamethasone suppression test.*

**Supplemental Table 3.** Differences between patients who changed over time their secretion status [3A: from non-functional adrenal tumors (NFAT) at baseline to mild autonomous cortisol secretion (MACS) at the last follow-up visit (LFUV); 3B: from MACS at baseline to NFAT at LFUV) and patients who remained in the same category.

| **Supplemental Table 3A** | **Patients with NFAT at baseline** | | | **Women with NFAT at baseline** | | | **Men with NFAT at baseline** | | |
| --- | --- | --- | --- | --- | --- | --- | --- | --- | --- |
| **Characteristics** | **NFAT**  **at LFUV**  **N= 55** | **MACS**  **at LFUV**  **N= 16** | ***p***  ***value*** | **NFAT**  **at LFUV**  **N= 33** | **MACS**  **at LFUV**  **N= 12** | ***p***  ***value*** | **NFAT**  **at LFUV**  **N= 22** | **MACS**  **at LFUV**  **N= 4** | ***p***  ***value*** |
| **Hypertension**, N (%) | 41 (76%) | 12 (75%) | 0.94 | 24 (75%) | 10 (83%) | 0.56 | 17 (77%) | 2 (50%) | 0.26 |
| *Valid cases* | *54* | *16* |  | *32* | *12* |  | *22* | *4* |  |
| **Hyperglycemia**, N (%) | 26 (49%) | 9 (56%) | 0.61 | 12 (37%) | 6 (50%) | 0.45 | 14 (67%) | 3 (75%) | 0.74 |
| *Valid cases* | *53* | *16* |  | *32* | *12* |  | *21* | *4* |  |
| **Dyslipidemia**, N (%) | 24 (48%) | 12 (75%) | 0.06 | 14 (45%) | 8 (67%) | 0.21 | 10 (53%) | 4 (100%) | 0.08 |
| *Valid cases* | *50* | *16* |  | *31* | *12* |  | *19* | *4* |  |
| **Bone impairment**, N (%) | 22 (63%) | 8 (67%) | 0.81 | 17 (65%) | 6 (69%) | 0.76 | 5 (55%) | 2 (100%) | 0.24 |
| *Valid cases* | *35* | *12* |  | *26* | *10* |  | *9* | *2* |  |
|  |  | | |  | | |  | | |
| **Supplemental Table 3B** | **Patients with MACS at baseline** | | | **Women with MACS at baseline** | | | **Men with MACS at baseline** | | |
| **Characteristics** | **NFAT**  **at LFUV**  **N= 18** | **MACS**  **at LFUV**  **N= 47** | ***p***  ***value*** | **NFAT**  **at LFUV**  **N= 12** | **MACS**  **at LFUV**  **N= 27** | ***p***  ***value*** | **NFAT  at LFUV**  **N= 6** | **MACS**  **at LFUV**  **N= 20** | ***p***  ***value*** |
| **Hypertension**, N (%) | 11 (61%) | 40 (85%) | **0.04** | 8 (67%) | 23 (85%) | 0.19 | 3 (50%) | 17 (85%) | 0.07 |
| *Valid cases* | *18* | *47* |  | *12* | *27* |  | *6* | *20* |  |
| **Hyperglycemia**, N (%) | 7 (41%) | 27 (59%) | 0.22 | 6 (50%) | 16 (59%) | 0.59 | 1 (20%) | 11 (58%) | 0.13 |
| *Valid cases* | *17* | *46* |  | *12* | *27* |  | *5* | *19* |  |
| **Dyslipidemia**, N (%) | 8 (50%) | 28 (62%) | 0.39 | 6 (55%) | 16 (64%) | 0.59 | 2 (40%) | 12 (60%) | 0.42 |
| *Valid cases* | *16* | *45* |  | *11* | *25* |  | *5* | *20* |  |
| **Bone impairment**, N (%) | 10 (91%) | 22 (71%) | 0.18 | 8 (89%) | 16 (84%) | 0.74 | 2 (100%) | 6 (50%) | 0.19 |
| *Valid cases* | *11* | *31* |  | *9* | *19* |  | *2* | *12* |  |
